# Risk of cardiocerebrovascular diseases is increased in Korean women with polycystic ovary syndrome: a nationwide cohort study

**DOI:** 10.1038/s41598-023-50650-y

**Published:** 2024-01-11

**Authors:** Ki-Jin Ryu, Hyuntae Park, Min Sun Kim, Hye Gyeong Jeong, Tak Kim

**Affiliations:** 1 Department of Obstetrics and Gynecology, Korea University College of Medicine, 73 Goryeodae-Ro, Seongbuk-Gu, Seoul, 02841 Republic of Korea; 2 Department of Biostatistics, Korea University College of Medicine, 73 Goryeodae-Ro, Seongbuk-Gu, Seoul, 02841 Republic of Korea

**Keywords:** Endocrine system and metabolic diseases, Reproductive disorders

## Abstract

To investigate the relationship between polycystic ovary syndrome (PCOS) and risk of cardiocerebrovascular disease in Korean women. This longitudinal cohort study using data from the Korean National Health Insurance Service included the women aged 15–44 years diagnosed with PCOS between 2002 and 2019, and the controls were matched 1:3 by age group, income, and region of residence. The endpoint outcomes of this study were the occurrence of ischemic heart disease, cerebrovascular diseases, and combined cardiocerebrovascular diseases in the PCOS and control groups. A stratified Cox proportional hazards regression analysis for matched data was performed to evaluate the relative hazard of events in the PCOS group compared to that in the control group. Among a total of 549,400 participants in the cohort, 137,416 women had a diagnosis of PCOS and 412,118 women did not have it. During a median follow-up of 54 months (interquartile range, 30–78 months), the incidence rates of all cardiovascular, ischemic heart, and cerebrovascular diseases were 6.6, 4.0, and 2.9, respectively, per 1000 person-years for women with PCOS, and 4.8, 2.8, and 2.3, respectively, per 1000 person-years for healthy control women. Women with PCOS had a higher hazard ratio of 1.224 (95% confidence interval, 1.18–1.27) of the composite cardiocerebrovascular diseases than those in the controls after propensity score matching for confounding variables, including body mass index, diabetes mellitus, hypertension, dyslipidemia, physical exercise level, alcohol consumption, current smoking, systolic and diastolic blood pressures, total cholesterol, and triglyceride levels. Hazard ratio for ischemic heart and cerebrovascular diseases was higher in women with PCOS than in the control group (hazard ratio, 1.254; 95% confidence interval, 1.20–1.31 and hazard ratio, 1.201; 95% confidence interval, 1.14–1.27, respectively). PCOS is associated with an increased risk of cardiocerebrovascular diseases in Korean women irrespective of their obesity. Counselling on the management of long-term risk of cardiovascular diseases should be offered to women with PCOS in East Asian countries where PCOS is characterized by a relatively low BMI.

## Introduction

Polycystic ovary syndrome (PCOS) is the most common endocrine disorder among women of reproductive age, affecting 8–13% of this population, and has been suggested as a risk factor for cardiometabolic disorders^1^. PCOS is characterized by typical features, including clinical or biochemical hyperandrogenism, ovulatory dysfunction, and polycystic ovaries^2^. Excessive androgen production has been assumed to play a major role in the pathophysiology of PCOS, and it also contributes to an increase in visceral adiposity, insulin resistance, and hyperinsulinemia^3^. The major consequences of these metabolic abnormalities, together with intrinsic hyperandrogenemia, are associated with a greater risk of several cardiometabolic disorders in women with PCOS, including obesity, type 2 diabetes mellitus (DM), dyslipidemia, hypertension, metabolic syndrome, and cardiovascular diseases (CVD)^4–6^. The increasing global prevalence of metabolic disorders has led to an association between PCOS and CVD risk, which is the leading cause of death in women receiving particular attention^7^. The latest recommendations from an international evidence-based guideline for the management of PCOS emphasized the screening practice for CVD risk, including regular monitoring of weight changes and excess weight^8^.

Although accumulated evidence indicates an association between PCOS and CVD risk factors, the relationship between PCOS and an increased long-term risk of CVD events remains controversial. It is unclear whether the association between PCOS and CVD is mediated by traditional risk factors or is independently associated with increased risk^6^. In particular, the overwhelming impact of obesity in driving traditional CVD risk factors is evident, as most women with PCOS in previous studies were overweight or obese^9^. A few previous review articles with meta-analysis reported that PCOS was associated with an increased risk of subsequent CVD events, including coronary heart diseases and cerebrovascular diseases^10^. However, it is noteworthy that most of these studies were conducted in Western populations and there is still a lack of data on Asian women to support the association between PCOS and CVD. East Asian women with PCOS have a low body mass index (BMI) and mild hirsutism compared to Western women with PCOS^8,11^. The long-term CVD risk in East Asian women diagnosed with PCOS, especially in those without obesity, has not been clearly established.

Thus, this study aimed to evaluate the association between PCOS and subsequent CVD risks in Korean women of reproductive age using nationwide population-based data.

## Materials and methods

### Data source

This retrospective matched cohort study used data from the Korea National Health Insurance Service (K-NHIS), a single medical insurer in the Republic of Korea managed by the government. The majority of Koreans (97.1%) are mandatory subscribers, and the database is freely accessible to medical researchers. It covers health insurance claims filed between January 1, 2002 and July 31, 2019. This database contains longitudinal information, including the participants’ demographics, as well as medical records, including disease code records according to the International Classification of Disease, Tenth Revision (ICD-10), use of inpatient and outpatient services, medical procedures, and mortality data. The study protocol was approved by the Institutional Review Board (IRB) of the Korea University Anam Hospital (IRB No:2020AN0298), which waived the requirement for informed consent. The National Health Information Data Request Review Committee approved the study protocol and use of NHIS data for research (NHIS-2021–1-529). This study was conducted in accordance with the principles of the Declaration of Helsinki.

### Study population

**Figure 1:**
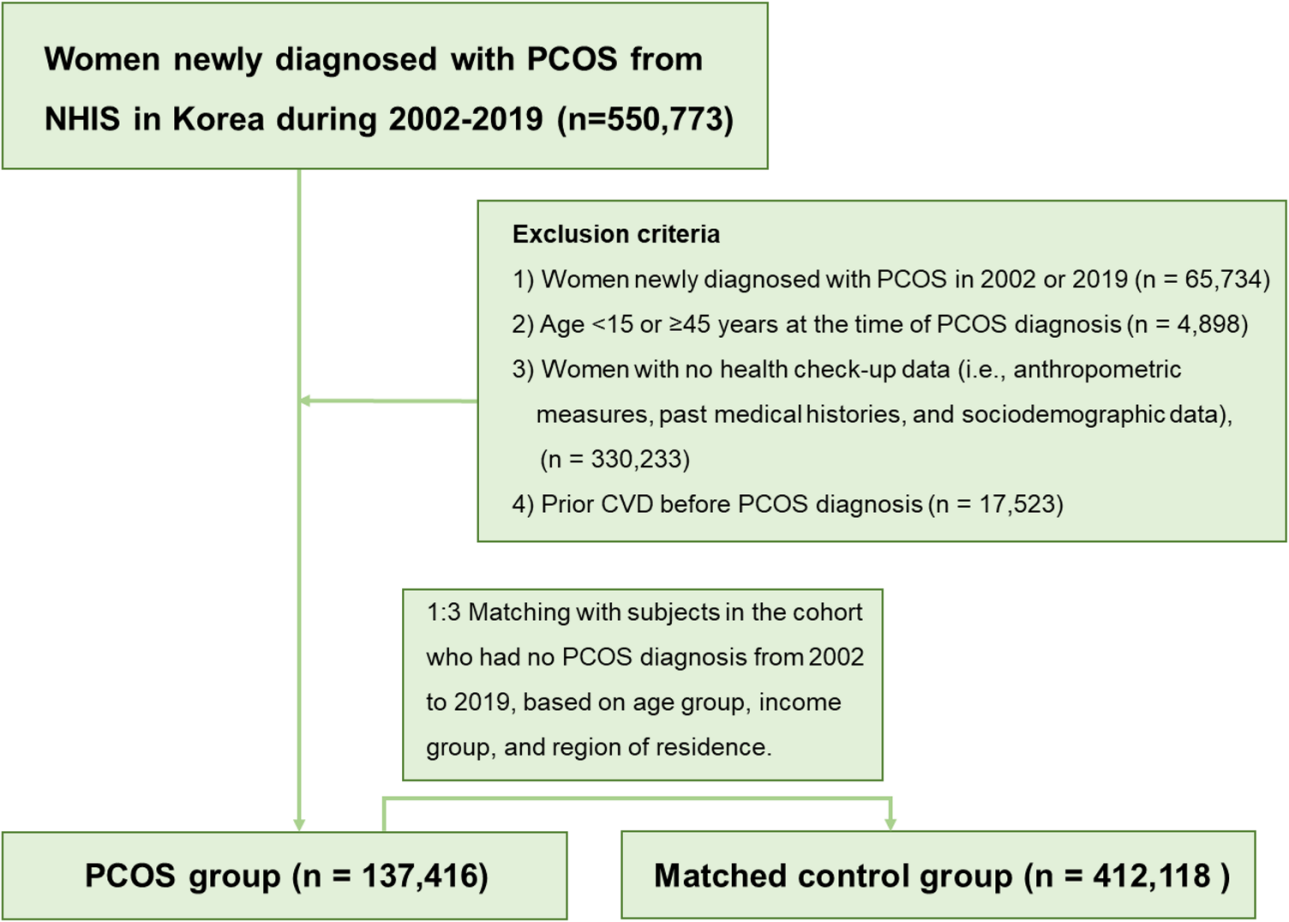
Flow chart of this study.

Diagnosis of PCOS requires an inpatient or outpatient record of ICD-10 code E282 in the database. Inclusion criteria were as follows: 1) women newly diagnosed with PCOS between January 2003 and December 2018, with the application of a 1-year washout period; 2) age 15–44 years at the time of PCOS diagnosis; and 3) available data on anthropometric measures, past medical histories, and sociodemographic data from health check-ups. Women who had been diagnosed with ischemic heart disease (ICD-10 code I20–25) or cerebrovascular diseases (ICD10 code I60–64) before PCOS diagnosis were excluded. These PCOS patients were matched 1:3 with subjects in the cohort who had no PCOS diagnosis between 2002 and 2019 (the control group). Matching was performed based on the age group, income group, and region of residence by greedy matching (three controls per case) using a nearest-neighbor algorithm. Greedy matching is a valid matching method used in observational studies. It sorts the cases and controls randomly and matches each case repeatedly with the closest controls until all cases are matched. The following age groups were defined: 15–19, 20–24, 25–29, 30–34, 35–39, and 40–44 years old. The income groups were categorized into 11 classes: one health aid class (class 0) and 10 employment health insurance classes divided by income quintile (class 1 [lowest income]–class 11 [highest income]), as described in the previous articles^5^. The region of residence was divided into 16 areas based on administrative districts. The regions were regrouped into urban (Seoul, Busan, Daegu, Incheon, Gwangju, Daejeon, and Ulsan) and rural (Gyeonggi, Gangwon, Chungcheongbuk, Chungcheongnam, Jeollabuk, Jeollanam, Gyeongsangbuk, Gyeongsangnam, and Jeju) groups. To prevent selection bias when choosing matched participants, the potential control group participants were sorted using a random number order and then selected from top to bottom. Finally, 1:3 matching resulted in the inclusion of 137,416 patients with PCOS and 412,118 control participants (Fig. 1).

### Main outcome measures and confounder variables

The endpoint outcomes of this study were the occurrence of ischemic heart disease (ICD-10 code I20–25), cerebrovascular diseases (ICD10 code I60–64), and combined cardiocerebrovascular diseases (ICD10 code I20–25 and I60–64) in the PCOS and control groups. Each patient was followed up from the index date until the earliest occurrence of any study outcome, death, or the end of the study period (July 31, 2019). Confounder variables measured for this study included patient age at the index date, BMI, medical history of diabetes mellitus (ICD-10 codes E11), hypertension (ICD-10 codes I10–15), dyslipidemia (ICD-10 codes E78), physical exercise level, alcohol consumption, current smoking, systolic and diastolic blood pressure (BP), total cholesterol, and triglyceride levels. The BMI was categorized into four groups based on the Asia–Pacific cutoff points: underweight (< 18.5 kg/m^2^), normal weight (18.5–22.9 kg/m^2^), overweight (23–24.9 kg/m^2^), and obese (≥ 25 kg/m^2^). Physical exercise level was categorized according to the frequency of activity lasting at least 20 min per day as follows: none, 1–4 times per week, and more than five times per week. The alcohol consumption level was classified into three groups as follows: none, mild to moderate (> 0 g to < 30 g/day), and heavy (≥ 30 g/day).

### Statistical analyses

Continuous variables are indicated as mean ± standard deviation (SD) and were compared using the paired *t*-test. Categorical variables are presented as numbers with percentages and compared using the chi-square test or McNemar test. The incidence per 1,000 person-years for the study outcome (number of cardiocerebrovascular disease events per 1,000 person-years) was calculated from the PCOS and control groups and their subgroups at the BMI level. Propensity score matching (PSM) and inverse probability of treatment weighting (IPTW) were used to reduce the effects of confounders between the PCOS and control groups. The propensity score defining each individual’s probability of having PCOS was developed using a multiple logistic regression model that included the following selected confounding variables: age, BMI, diabetes mellitus, hypertension, dyslipidemia, systolic and diastolic BPs, total cholesterol, triglyceride levels, history of alcohol consumption, current smoking, and physical exercise level. The baseline characteristics between the two groups were compared using generalized mixed models with appropriate link functions in each matched cohort. A stratified Cox proportional hazards regression analysis for matched data was performed to evaluate the relative hazard of events in the PCOS group compared to that in the control group after adjusting for covariates used in the multiple logistic regression model. We also used IPTW, which is a powerful tool for obtaining observational data^12^. The weights based on each individual’s propensity score were calculated by the inverse of the score in the PCOS group and the inverse of 1 minus the score in the control group^13^. We then estimated the hazard ratio (HR) and 95% CI from weighted Cox proportional hazards regression analysis using IPTW. Kaplan–Meier curves for the main outcome events were used to compare survival times between the PCOS and control groups and were tested using the log-rank test. For subgroup analyses, we divided the participants into obese (BMI ≥ 25 kg/m^2^) and non-obese (BMI < 25 kg/m^2^) groups, and stratified multivariate Cox regression analyses were performed within each subgroup. Statistical significance was defined as a two-sided *P*-value < 0.05. All statistical analyses were performed using the SAS software (version 9.4; SAS Institute Inc., Cary, NC, USA).

### Ethics approval

The study protocols were approved by the official review committee of the Korean government and Institutional Review Board of Korea University Anam Hospital (IRB No:2020AN0298).

#### Attestation statements

Data regarding any of the subjects in the study has not been previously published unless specified. Data will be made available to the editors of the journal for review or query upon request.

## Results

Among a total of 549,400 participants in the cohort, 137,416 women had a diagnosis of PCOS (PCOS groups) and 412,118 women did not have it (control group) during the studied periods. A comparison of baseline characteristics between the two groups is presented in Table 1. Women with PCOS were more frequently obese, had chronic diseases, including hypertension, diabetes, and dyslipidemia, had worse lipid profiles, higher BP, higher fasting glucose levels, and higher liver enzyme levels than women in the control group.

**Table 1:** Comparison of baseline characteristics between the PCOS and control groups after 1:3 matching by age group, income group, and region of residence in the Korean National Health Insurance Service data between 2002 and 2019.

	PCOS (n = 137,416)	Control (n = 412,118)	*P* value
Age	30.4 ± 5.5	30.4 ± 5.5	< 0.0001^a^
BMI (kg/m^2^)	22.3 ± 4.2	21.6 ± 3.5	< 0.0001^a^
BMI group (%)			< 0.0001^b^
Underweight (< 18.5 kg/m^2^)	12.8	13.9	
Normal (18.5–22.9 kg/m^2^)	54.2	60.0	
Overweight (23.0–24.9 kg/m^2^)	12.2	12.1	
Obese (≥ 25.0 kg/m^2^)	20.8	14.0	
Hypertension (%)	5.1	3.2	< 0.0001^c^
DM (%)	7.1	3.7	< 0.0001^c^
Dyslipidemia (%)	21.4	15.6	< 0.0001^c^
Physical exercise level (%)			< 0.0001^b^
None	17.6	18.2	
1–4 times/week	48.9	48.3	
≥ 5 times/week	33.5	33.6	
Alcohol consumption (%)			< 0.0001^b^
None	46.0	47.4	
mild to moderate (> 0 g to < 30 g/day)	52.9	51.6	
Heavy (≥ 30 g/day)	1.2	1.0	
Smoking (%)			< 0.0001^b^
Never	86.8	89.1	
Former smoker	5.8	4.6	
Current smoker	7.4	6.3	
Systolic BP (mmHg)	112.2 ± 12.2	111.5 ± 11.7	< 0.0001^a^
Diastolic BP (mmHg)	70.6 ± 9.1	70.0 ± 8.7	< 0.0001^a^
Fasting glucose (mg/dL)	90.1 ± 15.5	89.2 ± 13.6	< 0.0001^a^
Total cholesterol (mg/dL)	185.8 ± 34.3	180.8 ± 32.8	< 0.0001^a^
HDL cholesterol (mg/dL)	64.4 ± 18.9	64.9 ± 17.7	< 0.0001^a^
LDL cholesterol (mg/dL)	104.8 ± 115.9	101.7 ± 121.6	< 0.0001^a^
Triglycerides (mg/dL)	94.3 ± 71.0	81.7 ± 54.2	< 0.0001^a^
AST (IU/L)	21.4 ± 17.6	19.6 ± 15.5	< 0.0001^a^
ALT (IU/L)	19.1 ± 25.9	15.6 ± 18.6	< 0.0001^a^
GGT (IU/L)	21.0 ± 25.3	17.6 ± 18.7	< 0.0001^a^

Data are expressed as mean ± standard deviation, unless specified otherwise. ^a^Paired *t*-test, ^b^ chi-square test, and ^c^ McNemar test.

PCOS: polycystic ovary syndrome, IQR: interquartile range, DM: diabetes mellitus, BMI: body mass index, BP: blood pressure, HDL: high-density lipoprotein, LDLP: low-density lipoprotein, AST: aspartate aminotransferase, ALT: alanine transaminase, GGT: gamma-glutamyl transferase.

During a follow-up of 4.0 and 4.5 years for the PCOS and control groups, respectively, the incidence rate of all cardiocerebrovascular diseases was 6.6/1,000 and 4.8/1,000 person-years (*P* < 0.0001) in the PCOS and control groups, respectively. In particular, the incidence rates of ischemic heart disease and cerebrovascular disease were 4.0/1,000 and 2.9/1,000 person-years, respectively, in the PCOS group, whereas those in the control group were 2.8/1,000 and 2.3/1,000 person-years, respectively (*P* < 0.0001, Table 2).

**Table 2:** Comparison of the incidence of cardiovascular diseases between PCOS and control groups after 1:3 matching by age group, income group, and region of residence in the Korean National Health Insurance Service data between 2002 and 2019.

	PCOS (n = 137,416)	Control (n = 411,984)	*P* value
Follow-up years, median (IQR)	4.0	4.5	< 0.0001
Incidence per 1000 person-years of ischemic heart diseases (I20–25, %)	4.0	2.8	< 0.0001
Incidence per 1000 person-years of cerebrovascular diseases (I60–69, %)	2.9	2.3	< 0.0001
Incidence per 1,000 person-years of combined cardiocerebrovascular diseases (I20–25 & I60–69, %)	6.6	4.8	< 0.0001

PCOS: polycystic ovary syndrome, IQR: interquartile range.

**Figure 2:**
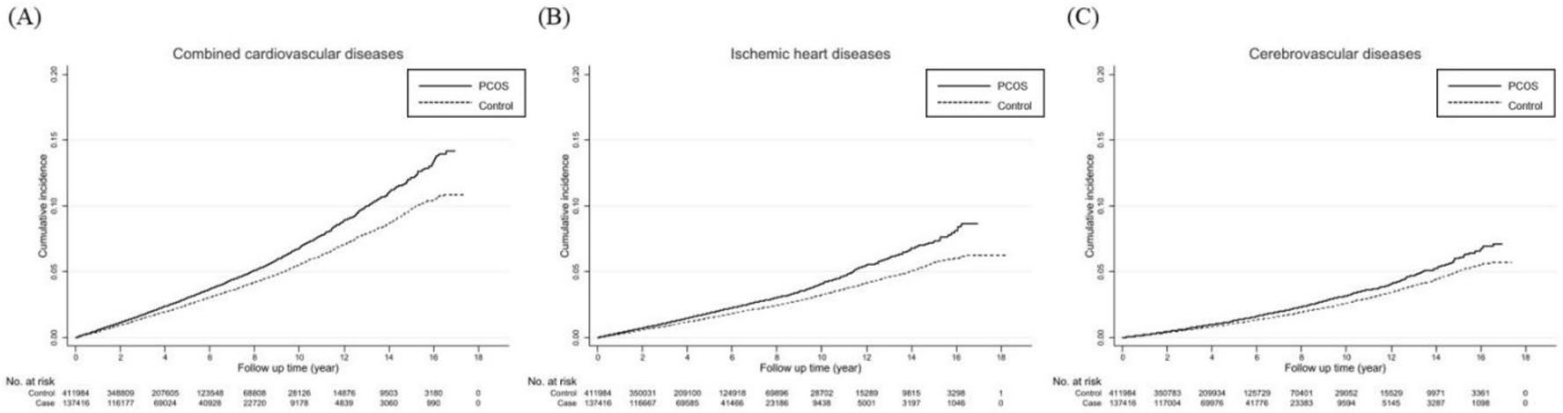
Kaplan–Meier survival curve analysis for the hazard of cardiovascular diseases in the PCOS and control groups (1:3 matching by age group, income group, and region of residence) from the Korean National Health Insurance Service data between 2003 and 2012, calculated after propensity score matching for confounding variables, including age, BMI, diabetes mellitus, hypertension, dyslipidemia, systolic BP, diastolic BP, total cholesterol, triglyceride levels, history of alcohol consumption, current smoking, and physical exercise level. PCOS: polycystic ovary syndrome, BMI: body mass index, BP: blood pressure.

The Kaplan–Meier survival curve shows the difference in the HR of ischemic heart diseases, cerebrovascular disease, and combined cardiocerebrovascular diseases between the PCOS and control groups (Fig. 2).

The results of the multivariate Cox regression analyses are presented in Table 3. After PSM for confounding variables, women with PCOS were found to have a higher HR (1.2 [95% CI, 1.2–1.3]) for cardiocerebrovascular diseases than women without PCOS. Subgroup analysis showed that PCOS was significantly associated with an increased HR for cardiocerebrovascular diseases in both obese and non-obese women (Table 3).

**Table 3:** Results of multivariate Cox regression analyses to determine HRs of cardiovascular diseases in women with PCOS compared to the matched control group after propensity score matching for confounding variables^a^ in the Korean National Health Insurance Service data from 2002 to 2019.

		Ischemic heart diseases (I20–25)		
		Entire cohort	Non-obese group (BMI < 25)	Obese group (BMI ≥ 25)
PCOS	Yes	1.3 (1.2–1.3)	1.2 (1.2–1.3)	1.3 (1.2–1.4)
		Cerebrovascular diseases (I60–69)		
		Entire cohort	Non-obese group (BMI < 25)	Obese group (BMI ≥ 25)
PCOS	Yes	1.2 (1.1–1.3)	1.2 (1.1–1.3)	1.2 (1.1 – 1.3)
		Combined cardiocerebrovascular diseases (I20–25 & I60–69)		
		Entire cohort	Non-obese group (BMI < 25)	Obese group (BMI ≥ 25)
PCOS	Yes	1.2 (1.2–1.3)	1.2 (1.1–1.2)	1.3 (1.2–1.3)

Data are presented as hazard ratios (90% confidence intervals). Statistical analyses were performed using stratified Cox proportional hazard regression analysis.

^a^Age, BMI, diabetes mellitus, hypertension, dyslipidemia, systolic BP, diastolic BP, total cholesterol, and triglyceride levels, and history of alcohol consumption, current smoking, and physical exercise level.

PCOS: polycystic ovary syndrome, BMI: body mass index, BP: blood pressure, HRs: hazard ratios.

## Discussion

This longitudinal study using nationwide population-based cohort data with an 18-year total studied duration revealed that women of reproductive age with PCOS have an approximately 1.3-fold higher risk of developing CVD than those without PCOS, even after controlling for several confounding factors, including BMI. It is important to note that most of our participants did not have obesity (BMI < 25 kg/m^2^) even in the PCOS group. The association between PCOS and increased HR for CVD remained significant in the obese and non-obese subgroups.

A recent umbrella review concluded that women with PCOS had a 1.3-fold greater risk of developing composite CVD than women without PCOS^10^, which was comparable to the results of our present study. More recently, a systematic review and meta-analysis including nine prospective and 14 retrospective cohort studies found that women with PCOS did not have an increased risk of coronary disease events (RR 1.78, [0.99–3.23]), whereas they had an increased risk of hypertension (RR, 1.75 [1.42–2.15]), type 2 diabetes (RR 3.0 [2.56–3.51]), and non-fatal cerebrovascular disease events (RR 1.41 [1.02–1.94])^14^. It is noteworthy that most studies included in those meta-analyses were conducted in Western populations, mostly involved Caucasian women, and there was a lack of data on Asian women^14–16^. To the best of our knowledge, this is the first longitudinal population-based study to reveal an association between PCOS and the incidental CVD risk in Korean women. Our findings should be considered in counselling the Asian women with PCOS and offering the guidelines for the management of long-term cardiovascular health among those population.

Accumulated evidence has indicated the association between PCOS and subclinical CVD, as measured by various cardiovascular factors, including flow-mediated dilation, arterial stiffness, pulse wave velocity, carotid intima-media thickness, visceral and epicardial fat thickness, and coronary artery calcium^17^. The exact mechanism underlying this association remains unclear and seems to be complex and multifactorial, including factors like insulin resistance, hyperinsulinemia, hyperandrogenism, obesity, inflammation, and metabolic syndrome^17–19^. In particular, hyperandrogenism affects approximately 75% of women with PCOS and is a contributor to endothelial dysfunction, which has a substantial role in the pathogenesis of atherosclerosis and other CVDs^17,20^. Furthermore, hyperandrogenism also contributes to an increase in insulin resistance and visceral adiposity^3^.

Both obesity and PCOS are linked to a higher CVD risk; according to previous studies, more than half of women with PCOS were obese. However, only 20% of women with PCOS were obese in our study, and the risk of CVD increased in obese and non-obese women with PCOS after adjusting for confounders, including BMI. Our findings suggest that obesity alone does not explain the incremental risk of PCOS in Asian women. However, visceral fat adiposity and chronic low-grade inflammation can be observed in women with PCOS, regardless of general obesity^21–23^. Furthermore, recent studies have identified the hypertriglyceridemic waist-to-height ratio as a closely reflective indicator of cardiometabolic disorders in the elderly. Nevertheless, there is currently no available information concerning the significance of these indicators in women with PCOS^24^. Further studies should investigate the complex interaction of these factors together with intrinsic hyperandrogenism and insulin resistance in women with PCOS to understand how the risk of CVD truly increases in those populations.

The latest recommendations from an international guideline for PCOS specifically address the screening practice for CVD risk: regular monitoring of weight changes and excess weight, fasting lipid profile measurement in overweight and obese women with PCOS, and assessment of traditional CVD risk factors, including obesity, smoking, dyslipidemia, hypertension, impaired glucose tolerance, and lack of physical activity, although these recommendations were mainly from clinical consensus, rather than from adequate evidence on PCOS^8^. However, surveys conducted in Western countries indicate that patients are dissatisfied with the counselling they receive related to the screening and management of their CVD risk^25^. In Korea, this problem seems more severe due to the lack of data on Asian women with PCOS who were overweight or obese much less frequently than Western women^5,11^. A recent review article comprehensively organized CVD risk screening and preventive strategies to reduce CVD risk in women with PCOS^6^, and more constructive efforts are needed in real clinical fields based on such well-organized recommendations and accumulating evidence.

To the best of our knowledge, this is the first population-based longitudinal study to reveal the increased risk of CVD events in Asian women with PCOS, independent of confounders, including obesity. The main strengths of this study include the large size of the study population, representativeness of Korean women, and adjustment for important confounding factors, including various medical histories and anthropometric, laboratory, and socioeconomic measures, which were offered by the NHIS databases. However, our study has some limitations. First, the incidence of PCOS may be underestimated in the NHIS database because it was estimated based on physicians’ diagnoses of the disease, and because asymptomatic patients are less likely to visit a gynecological clinic than symptomatic or high-risk patients. However, both the high accessibility of health services and the frequency of anovulatory symptoms of PCOS in South Korea might compensate for this limitation. Second, women with features of PCOS, such as irregular menstruation and hirsutism, could not be excluded from the control group because the data on these parameters were not available in the NHIS database. Third, this study could not assess the diagnostic details of PCOS due to the characteristics of the claims database; therefore, it could not assess the CVD risks according to the different phenotypes of PCOS^26^. Although CVD risk reportedly increases regardless of the phenotypes in women with PCOS^27^, further studies that include these measures may be needed to understand the exact mechanisms underlying the association between PCOS and CVD risk. Fourth, information about combined oral contraceptives (COCs) use was not available in the NHIS database because all COCs are prescribed as non-reimbursement medication in South Korea. COCs are the most popular treatment options for PCOS and at the same time an important risk factor for CVD^28^. Furthermore, despite making adjustment for several variables, we were still unable to incorporate certain factors into the analysis, such as medical history variables like the use of hypolipidemic drugs, lifestyle factors like sleep, and psychological variables. Further studies that include such information are warranted to confirm our findings.

## Conclusions

Our nationwide population-based longitudinal study revealed that women diagnosed with PCOS have a higher HR for CVD than those without PCOS, irrespective of the presence of obesity. Counselling on the management of long-term risk of CVD should be offered not only to obese women with PCOS but also to non-obese women with PCOS in East Asian countries where PCOS is characterized by a relatively low BMI. Our findings may be helpful in determining guidelines for the management of PCOS in East Asian women and in guiding physicians in treating PCOS patients with healthy body weight.

### Supplementary Information

Supplementary Information.

## Data Availability

The datasets used and/or analyzed during the current study available from the corresponding author on reasonable request.

## References

[1] Ehrmann DA (2005). Polycystic ovary syndrome. N. Engl. J. Med..

[2] Rotterdam EA-SPCWG (2004). Revised 2003 consensus on diagnostic criteria and long-term health risks related to polycystic ovary syndrome. Fertil. Steril..

[3] Christakou CD, Diamanti-Kandarakis E (2008). Role of androgen excess on metabolic aberrations and cardiovascular risk in women with polycystic ovary syndrome. Womens Health (Lond).

[4] Osibogun O, Ogunmoroti O, Michos ED (2020). Polycystic ovary syndrome and cardiometabolic risk: Opportunities for cardiovascular disease prevention. Trends Cardiovasc. Med..

[5] Ryu KJ, Kim MS, Kim HK, Kim YJ, Yi KW, Shin JH (2021). Risk of type 2 diabetes is increased in nonobese women with polycystic ovary syndrome: The national health insurance service-national sample cohort study. Fertil. Steril..

[6] Guan C, Zahid S, Minhas AS, Ouyang P, Vaught A, Baker VL (2022). Polycystic ovary syndrome: A "risk-enhancing" factor for cardiovascular disease. Fertil. Steril..

[7] Harvey RE, Coffman KE, Miller VM (2015). Women-specific factors to consider in risk, diagnosis and treatment of cardiovascular disease. Womens Health (Lond).

[8] Teede HJ, Misso ML, Costello MF, Dokras A, Laven J, Moran L (2018). Recommendations from the international evidence-based guideline for the assessment and management of polycystic ovary syndrome. Fertil. Steril..

[9] Dokras A (2022). Heart health in polycystic ovary syndrome: Time to act on the data. Fertil Steril.

[10] Okoth K, Chandan JS, Marshall T, Thangaratinam S, Thomas GN, Nirantharakumar K (2020). Association between the reproductive health of young women and cardiovascular disease in later life: Umbrella review. BMJ.

[11] Kim MJ, Lim NK, Choi YM, Kim JJ, Hwang KR, Chae SJ (2014). Prevalence of metabolic syndrome is higher among non-obese PCOS women with hyperandrogenism and menstrual irregularity in Korea. PLoS One.

[12] Curtis LH, Hammill BG, Eisenstein EL, Kramer JM, Anstrom KJ (2007). Using inverse probability-weighted estimators in comparative effectiveness analyses with observational databases. Med. Care.

[13] Mansournia MA, Altman DG (2016). Inverse probability weighting. BMJ.

[14] Wekker V, van Dammen L, Koning A, Heida KY, Painter RC, Limpens J (2020). Long-term cardiometabolic disease risk in women with PCOS: A systematic review and meta-analysis. Hum. Reprod. Update.

[15] Zhao L, Zhu Z, Lou H, Zhu G, Huang W, Zhang S (2016). Polycystic ovary syndrome (PCOS) and the risk of coronary heart disease (CHD): A meta-analysis. Oncotarget.

[16] Zhou Y, Wang X, Jiang Y, Ma H, Chen L, Lai C (2017). Association between polycystic ovary syndrome and the risk of stroke and all-cause mortality: Insights from a meta-analysis. Gynecol. Endocrinol..

[17] Gomez JMD, VanHise K, Stachenfeld N, Chan JL, Merz NB, Shufelt C (2022). Subclinical cardiovascular disease and polycystic ovary syndrome. Fertil. Steril..

[18] Cooney LG, Dokras A (2018). Beyond fertility: Polycystic ovary syndrome and long-term health. Fertil. Steril..

[19] van der Ham K, Louwers YV, Laven JSE (2022). Cardiometabolic biomarkers in women with polycystic ovary syndrome. Fertil. Steril..

[20] Usselman CW, Yarovinsky TO, Steele FE, Leone CA, Taylor HS, Bender JR (2019). Androgens drive microvascular endothelial dysfunction in women with polycystic ovary syndrome: Role of the endothelin B receptor. J. Physiol..

[21] Delitala AP, Capobianco G, Delitala G, Cherchi PL, Dessole S (2017). Polycystic ovary syndrome, adipose tissue and metabolic syndrome. Arch. Gynecol. Obstet..

[22] Rudnicka E, Suchta K, Grymowicz M, Calik-Ksepka A, Smolarczyk K, Duszewska AM (2021). Chronic low grade inflammation in pathogenesis of PCOS. Int. J. Mol. Sci..

[23] Cascella T, Palomba S, De Sio I, Manguso F, Giallauria F, De Simone B (2008). Visceral fat is associated with cardiovascular risk in women with polycystic ovary syndrome. Hum. Reprod..

[24] Zhang P, Xiong Y, Chen M, Zhang H, Sun N, Wu F (2023). The relationship between hypertriglyceridemic wait-to-height ratio and hypertension–diabetes comorbidity among older adult. Front. Public Health.

[25] Gibson-Helm M, Teede H, Dunaif A, Dokras A (2017). Delayed diagnosis and a lack of information associated with dissatisfaction in women with polycystic ovary syndrome. J. Clin. Endocrinol. Metab..

[26] Lizneva D, Suturina L, Walker W, Brakta S, Gavrilova-Jordan L, Azziz R (2016). Criteria, prevalence, and phenotypes of polycystic ovary syndrome. Fertil. Steril..

[27] Daan NM, Louwers YV, Koster MP, Eijkemans MJ, de Rijke YB, Lentjes EW (2014). Cardiovascular and metabolic profiles amongst different polycystic ovary syndrome phenotypes: who is really at risk?. Fertil. Steril..

[28] Carmina E (2013). Oral contraceptives and cardiovascular risk in women with polycystic ovary syndrome. J. Endocrinol. Invest..

