# Supplementary material for: Risk of cardiocerebrovascular diseases is increased in Korean women with polycystic ovary syndrome: a nationwide cohort study
Source: Sci Rep. 2024 Jan 11;14:1055. doi: 10.1038/s41598-023-50650-y (PMC10784481; doi:10.1038/s41598-023-50650-y)
Supplement: Supplementary file 1 — Supplementary Information. [file 41598_2023_50650_MOESM1_ESM.docx]

**Supplementary Table.** Results of multivariate Cox regression analyses to determine HRs of cardiovascular diseases according to the age in women with PCOS compared to the matched control group after propensity score matching for confounding variables^a^.

| Ischemic heart diseases |  |  | Entire cohort | 15 ≤ age < 30 | 30 ≤ age < 45 |
| --- | --- | --- | --- | --- | --- |
|  | PCOS | Yes | 1.3 (1.2–1.3) | 1.1 (1.0–1.1) | 1.3 (1.3 – 1.4) |
| Cerebrovascular diseases |  |  | Entire cohort | 15 ≤ age < 30 | 30 ≤ age < 45 |
|  | PCOS | Yes | 1.2 (1.1–1.3) | 1.0 (0.9–1.1) | 1.2 (1.1 – 1.3) |
| Combined cardiocerebrovascular diseases |  |  | Entire cohort | 15 ≤ age < 30 | 30 ≤ age < 45 |
|  | PCOS | Yes | 1.2 (1.2–1.3) | 1.1 (1.0–1.1) | 1.2 (1.1–1.3) |

Data are presented as hazard ratios (90% confidence intervals). Statistical analyses were performed using stratified Cox proportional hazard regression analysis.

^a^Age, body mass index, diabetes mellitus, hypertension, dyslipidemia, systolic BP, diastolic BP, total cholesterol, and triglyceride levels, and history of alcohol consumption, current smoking, and physical exercise level.

Abbreviations: PCOS, polycystic ovary syndrome; BP, blood pressure; HRs, hazard ratios
